# Clinical and omics biomarkers in osteoarthritis diagnosis and treatment

**DOI:** 10.1016/j.jot.2024.12.007

**Published:** 2025-01-22

**Authors:** Muhai Deng, Cong Tang, Li Yin, Yunsheng Jiang, Yang Huang, Yong Feng, Cheng Chen

**Affiliations:** a College of Medical Informatics, Chongqing Medical University, Chongqing, 400016, China; b Department of Orthopaedics, General Hospital of Western Theater Command, Chengdu, 610083, China; c Department of Orthopedics, The First Affiliated Hospital of Chongqing Medical University, Chongqing, 400016, China; d State Key Laboratory of Trauma, Burns and Combined Injury, Department of Wound Infection and Drug, Daping Hospital, Army Medical University, Chongqing, 400042, China; e Department of Orthopedic Surgery, Chongqing Emergency Medical Center, Chongqing University Central Hospital, Chongqing, 400014, China

**Keywords:** Bioinformatics, Biomarkers, Cytokines, Early diagnosis, Multi-omics, Osteoarthritis

## Abstract

Osteoarthritis (OA) is a prevalent degenerative joint disease that significantly impacts the quality of life for hundreds of millions, and is a major cause of disability. Despite this, diagnostic and therapeutic options for OA are still limited. With advances in molecular biology, an increasing number of OA biomarkers have been identified, which not only enhances our understanding of OA pathogenesis, but also offers new approaches for OA diagnosis and treatment. This review discussed the research progress on traditional OA biomarkers, and analyzed the application of various omics, including genomics, transcriptomics, proteomics, and metabolomics, in the diagnosis and treatment of OA. Furthermore, we explored how integrating multi-omics methods can reveal interactions among different biomolecules and their roles in the development of OA. This emerging interdisciplinary approach not only provides a more comprehensive understanding of the fundamental biological characteristics of OA, but also aids in identifying new integrated biomarkers, thereby allowing for more accurate predictions of disease progression and treatment responses. The identification and development of biomarkers offer new perspectives in understanding OA, enhancing the specificity and sensitivity of biological diagnostic markers, providing a basis for the design of targeted drugs, and ultimately advancing the development of precision diagnosis and treatment strategies in clinical OA.

This study provides an overview of both commonly used and emerging biomarkers of OA which is beneficial for a more accurate, timely, effective clinical diagnosis and treatment for OA.

## Graphical abstract

**Figure:**
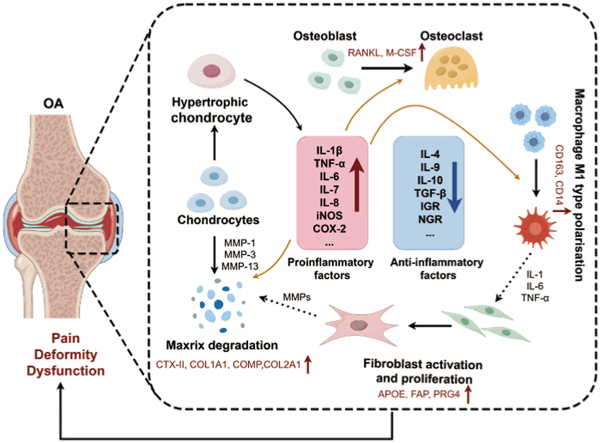

## Teaser

The Translational potential of this article: This study provides an overview of both commonly used and emerging biomarkers of OA which is beneficial for a more accurate, timely, effective clinical diagnosis and treatment for OA.

## 1 Introduction

Osteoarthritis (OA) is a prevalent degenerative joint disease that affects a significant portion of the global population [1]. It manifests as joint pain, swelling, and ultimately mechanical failure, impacting the entire joint, including cartilage, synovium, and subchondral bone. OA is characterized by progressive damage to the articular cartilage, joint space narrowing, subchondral bone remodeling, osteophyte formation, and synovitis [2]. Although early comprehensive assessment of OA can currently be achieved through the combination of patient symptoms, clinical signs, and imaging studies, the incidence and prevalence of OA remain difficult to quantify. This challenge is primarily due to the difficulty in detecting and diagnosing cartilage damage before the onset of noticeable symptoms [3]. According to the literature review, 25–75 % of knee pain cannot be diagnosed as OA through radiography [4]. Consequently, there is a growing focus on identifying biomarkers for OA, which provides insights into the biochemical changes occurring in joint tissues before structural damage becomes evident through traditional imaging techniques. Biomarkers for OA such as the C-terminal telopeptide of collagen type II (CTX-II), inflammatory cytokines, matrix metalloproteinases (MMPs), and cartilage oligomeric matrix protein (COMP) are secreted into bodily fluids due to metabolic activities in the joint tissues [5]. These biomarkers have been shown to be crucial in enabling timely therapeutic interventions and improving clinical outcomes for OA patients [6]. At the same time, advancements in omics technology have increased the likelihood of more comprehensive and in-depth explorations to discover OA biomarkers [7]. Omics research, including genomics, transcriptomics, proteomics, and metabolomics, has been widely applied in the search for disease biomarkers [8]. Further, integrating multi-omics data plays a crucial role in disease subtyping, biomarker prediction, and extracting valuable insights from large datasets, offering perspectives that might not be achievable with single-omics analysis [9]. Therefore, this review explores in detail the current progress of clinical and experimental studies on OA biomarkers, and further analyzes the latest developments of OA biomarkers in the fields of genomics, transcriptomics, proteomics, and metabolomics.

## 2 Materials and methods

This review discusses both common OA biomarkers and those found through omics analysis techniques, based on research from the early 2000s to May 2024. A search was performed using Google Scholar, PubMed, and ScienceDirect with keywords ‘Osteoarthritis’, ‘osteoarthritis’, ‘matrix metalloproteinases’, ‘cartilage oligomeric matrix protein’, ‘C-terminal telopeptide of collagen type II’, ‘biomarker’, ‘senescence’, ‘omics’, ‘genomics’, ‘genetics’, ‘transcriptomics’, ‘proteomics’, ‘metabolomics’, and ‘multiomics’. Studies including the following types of data (original, review, systematic, meta-analysis) were included and extracted.

## 3 Biomarkers (from clinical and experimental studies)

**Fig. 1:**
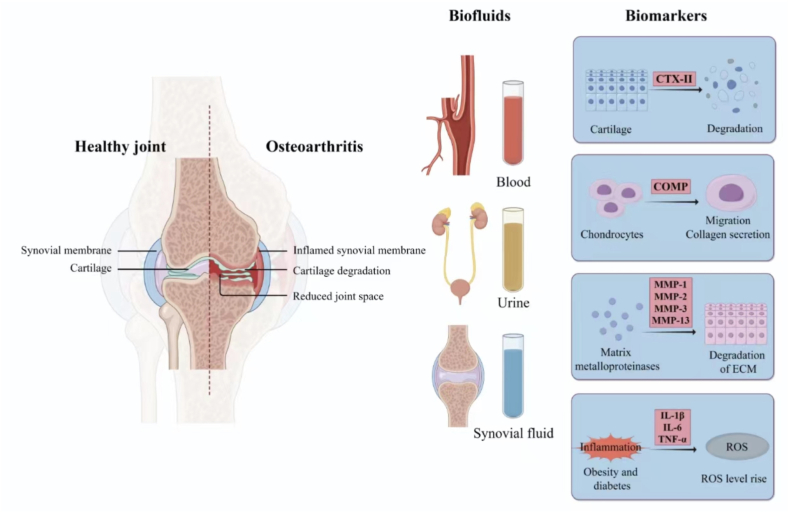
**A diagram of key biomarkers in body fluids of OA patients**. CTX-II, COMP, MMPs, and inflammatory cytokines have been studied as potential biomarkers for the diagnosis, progression, and prognosis of OA in various biological fluids, including blood (plasma and serum), synovial fluid, and urine. Figure created with the help of Figdraw (https://www.figdraw.com).

A prerequisite for developing effective and targeted early treatment methods is understanding the disease state at the molecular level [10]. OA biomarkers refer to molecules secreted into body fluids during the metabolism of joint cartilage, subchondral bone, and synovial tissue. These include extracellular matrix (ECM) components, such as CTX-II, COMP, MMPs, inflammatory cytokines, and other effector molecules (Fig. 1).

### 3.1 CTX-Ⅱ

Collagen fibers within articular cartilage are primarily composed of type II collagen (accounting for 90 %–95 %), and studies have shown a close relationship between changes in type II collagen and the progression of OA [11]. When cartilage is damaged, type II collagen is cleaved by proteases to produce CTX-II. CTX-II is then released into the joint cavity and serum, exhibiting high specificity and sensitivity for OA diagnosis. Compared to other cartilage metabolism biomarkers, CTX-II offers greater stability over time (>24 h at room temperature) and good reliability (able to withstand several freeze–thaw cycles while maintaining its properties). Derived entirely from mature, non-synthetic type II collagen with a small molecular weight, CTX-II can enter the bloodstream and is eventually excreted through urine [12]. Therefore, it is also the only biomarker that can be detected in blood, urine, and synovial fluid [13].

It has been shown that the concentration of CTX-II in the urine of OA patients is 1.53 times that of the control group. Particularly in knee OA, the level of CTX-II in urine is increased by 20 % [13]. Similarly, the average level of CTX-II in synovial fluid is higher than control group at all time intervals, peaking within a few hours after joint injury [12]. Considering that serum may exhibit less analytical and biological variability than urine, a serum CTX-II detection method was developed [14]. It is found that serum CTX-II levels have a stronger ability to distinguish between normal individuals and those with early knee OA compared to serum MMP-3, with an accuracy of 66.83 % [15]. Moreover, studies have shown that CTX-II can reflect the degree of articular cartilage damage and degradation, and it has a certain correlation with the progression of OA. In early diagnosis of OA using magnetic resonance imaging, X-rays, and several biomarkers such as COMP, CTX-I, and CTX-II, the level of CTX-II in the follow-up patients was significantly higher than the levels of other biomarkers after 3 months [16]. However, CTX-II also has some limitations. For instance, CTX-II shows varying degrees of sensitivity in detecting knee and hip OA. The standardized mean difference for detecting knee OA is 0.48 (95 % CI, 0.32, 0.64; P < 0.0001), while the standardized mean difference for detecting hip OA is 0.76 (95 % CI, 0.09, 1.42; P = 0.03) [17]. This variation in sensitivity can lead to inconsistencies in diagnostic accuracy, making it challenging to rely solely on CTX-II for early OA diagnosis. In conclusion, although CTX-II can reflect joint degeneration and cartilage deterioration before radiographic changes appear, it has certain limitations in terms of sensitivity, specificity, and its association with the late stages of the disease.

### 3.2 COMP

COMP, also known as thrombospondin-5, is a homologous pentameric EMC glycoprotein classified as a thrombospondin. It is a major component of non-collagenous cartilage protein, exhibiting strong cartilage tissue specificity [18]. It can bind to type II collagen fibers, playing a role in regulating collagen fiber remodeling and endochondral ossification [19]. When joint cartilage is damaged, the degradation of the cartilage matrix leads to an increase in COMP expression [20]. Therefore, COMP is a recognized biochemical marker associated with cartilage destruction.

COMP was initially considered a component of cartilage, with high expression in both developing and mature cartilage, as well as in tendons and ligament [21]. In a previous study investigating the release of COMP in the synovial fluid and blood of OA patients after joint injury, the COMP levels in the synovial fluid of the OA group were 47 μg/mL, which is higher than the reference levels in healthy knee joint volunteers. However, the COMP concentration in patients with advanced OA no longer increases [22]. Therefore, COMP is considered useful for monitoring treatment efficacy, disease progression, and repair in OA and other joint diseases. A study showed that serum COMP levels are statistically significant in patients with hip OA, but not with knee OA [23]. However, in another study of knee OA, serum COMP levels were measured in 150 subjects, results showed that OA patients have higher serum COMP levels than the control group, and the COMP levels were negatively correlated with disease duration and positively correlated with age, BMI, pain scores, and interleukin (IL)-1β. The findings suggest that serum COMP levels can serve as a diagnostic marker for knee OA [24]. A meta-analysis also found that serum COMP is significantly elevated in patients with knee OA compared to control group, indicating that serum COMP has the potential to distinguish between patients with knee OA and healthy subjects [25]. In studies of OA related to anterior cruciate ligament (ACL) defects, the serum COMP levels are significantly higher in the early OA group compared to the non-OA group. The optimal threshold for serum COMP is determined to be 152 ng/mL, suggesting that serum COMP can be used to detect early cartilage changes in patients with ACL defects [26]. However, COMP is not a specific biomarker for OA, as its levels are elevated in rheumatoid arthritis [27] and systemic sclerosis [28].

### 3.3 MMPs

MMPs are part of the zinc-dependent endopeptidase family. They can degrade ECM components, including collagen, laminin, fibronectin, hyaluronic acid, and proteoglycans. Degradation of the ECM by cleaving internal peptide bonds of target proteins is a characteristic of OA. Based on their structure and substrate specificity, MMPs can be classified into six categories: membrane-type MMPs (MT-MMPs), collagenases (MMP-1, MMP-13), gelatinases (MMP-2, MMP-9), stromelysins (MMP-3), matrilysins (MMP-7), and metalloelastases (MMP-12) [29]. Details are described in Table 1. Most MMPs are associated with ECM transformation and cartilage degradation in OA, including MMP-3, MMP-8, MMP-10, MMP-13, and MMP-14. Nonetheless, soluble collagenases MMP-1, MMP-8, and MMP-13 are essential for the degradation, with MMP-13 being predominant [30]. In murine OA models, MMP-13 levels are correlated with hypertrophic chondrocytes present in the early stages of OA, where its overexpression leads to ECM degradation and induces OA [31]. Conversely, in MMP-13 knockout mice, the development of OA is inhibited by protecting cartilage from proteoglycan loss and resultant damage [32]. Therefore, MMP-13 is considered particularly relevant to the degradation of articular cartilage due to its aggressive breakdown of type II collagen in OA. Moreover, in an immunohistochemical analysis of cartilage and subchondral bone from 51 patients, MMP-13 levels were found to be significantly higher in the severely worn medial tibial plateau cartilage compared to the relatively intact lateral cartilage [33]. However, although MMP-13 is primarily involved in the degradation of type II collagen, it also targets other matrix molecules, such as types I, III, IV, IX, and X collagen, perlecan, osteonectin, and proteoglycans. It may play a role in matrix turnover in healthy cartilage [34]. Apart from MMP-13, a study on MMP-3 levels in the blood and synovial fluid of OA patients found that MMP-3 expression is significantly higher in OA patients compared to the healthy group. Additionally, MMP-3 levels are positively correlated with the radiographic grading of the knee joint [35]. Similarly, an analysis of synovial fluid and peripheral blood samples from 39 obese female OA patients revealed that higher radiographic grading of OA correlated with increased MMP-3, MMP-9, and MMP-13 levels in serum and synovial fluid [36]. In summary, among the most classic collagenases (MMP-1, MMP-8 and MMP-13), MMP-13 can preferentially digest the main component of cartilage, type II collagen, as well as degrade proteoglycan aggregating proteins, thus playing a dual role in matrix destruction. Therefore, it is considered the most important MMP in the pathogenesis of OA and an attractive target for the development of OA therapeutic inhibitors [37].

**Table 1:** Functions and descriptions of MMP family members.

Gene	Name	Type	Location	Function	References
MMP-1	interstitial collagenase	collagenases	secreted	MMP-1 degrades interstitial collagens, type I, II, and III	[38]
MMP-2	gelatinase-A	gelatinases	secreted	MMP-2 can degrade proteoglycans, fibronectin, elastin, laminin, type I, IV, V, VII, and X collagens. MMP-2 promotes cell migration by releasing chemokines. MMP-2 is involved in the degradation of the ECM.	[39,40]
MMP-3	stromelysin-1	stromelysins	secreted	MMP-3 can also activate other MMPs, such as MMP-1, MMP-7, and MMP-9. MMP-3 can promote vascular growth into cartilage, facilitate the aggregation of inflammatory cells, and inhibit the differentiation of mesenchymal stem cells into chondrocytes.	[41,42]
MMP-7	matrilysin-1	matrilysin	secreted	MMP-7 can degrade ECM components, including laminin, elastin, tenascin-C, hyaluronic acid, fibronectin, types III, IV, V, IX, X, and XI collagen, types I, II, IV, and V gelatin, and proteoglycans. MMP-7 can cleave surface molecules of non-vascular endothelial cells. MMP-7 can also cleave and activate other MMPs, such as MMP-1, -2, and -9.	[43]
MMP-8	neutrophil collagenase	collagenases	secreted	MMP-8 can cleave type I, II, and III collagens.	[44]
MMP-9	gelatinase-B	gelatinases	secreted	MMP-9 can degrade the ECM, initiating and promoting the formation of new blood vessels.	[45]
MMP-10	stromelysin-2	stromelysin	secreted	MMP-10 regulates the collagenolytic activity of macrophage M2	[46]
MMP-11	stromelysin-3	stromelysin	secreted	MMP-11 can cleave collagen VI and non-structural ECM component substrates	[47]
MMP-12	metalloelastase	metalloelastase	secreted	MMP-12 can degrade type IV collagen, fibronectin, laminin, gelatin, proteoglycan, heparin, and chondroitin sulfate.	[48]
MMP-13	Collagenase-3	collagenases	secreted	MMP-13 can degrade type I, II, and III collagen, as well as other matrix molecules in cartilage, such as proteoglycans, type IV and IX collagen, osteonectin, and perlecan. MMP-13 can also degrade IGD-aggregated proteins and proteins in the ECM, and can cleave fibronectin.	[49]
MMP-14	MT1-MMP	Membrane-Type MMPs	Membrane-associated	MMP-14 is ubiquitously expressed and cleaves the ECM and non-matrix substrates. MMP-14 promotes LDLR cleavage, increases plasma LDL-C levels, and exacerbates the progression of atherosclerosis. MMP-14 is highly expressed in chondrocytes and synovial cells during arthritis and mediates the invasion of synovial fibroblasts.	[50]
MMP-15	MT2-MMP	Membrane-Type MMPs	Membrane-associated	MMP-15 can degrade Collagen I, II, III, gelatin, aggrecan, fibronectin, laminin, nidogen, perlecan, pro-MMP-2, pro-MMP-13, tissue transglutaminase, tenascin, and vitronectin.	[51]
MMP-16	MT3-MMP	Membrane-Type MMPs	Membrane-associated	MMP-16 can degrade Collagen I, II, III, gelatin, aggrecan, fibronectin, laminin, nidogen, perlecan, pro-MMP-2, pro-MMP-13, tissue transglutaminase, tenascin, and vitronectin.	[52]
MMP-17	MT4-MMP	Membrane-Type MMPs	Membrane-associated	MMP17 controls the integrity of the extracellular matrix aggregation proteins in articular cartilage under inflammatory conditions.	[53]

### 3.4 Inflammatory cytokines

**Fig. 2:**
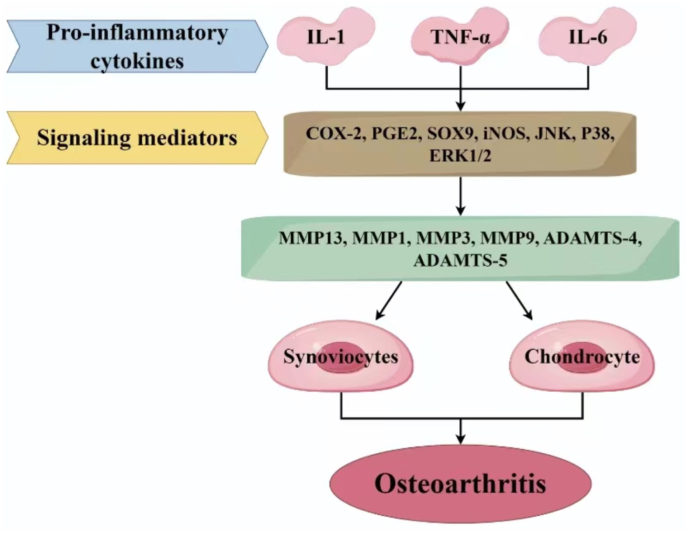
The major inflammatory factors IL-1, TNF-α, and IL-6 impact the progression of OA. IL-1, TNF-α, and IL-6 can induce the production of other cytokines, MMPs, and prostaglandins, while inhibiting the synthesis of proteoglycans and type II collagen. Activated chondrocytes also produce MMP-1, MMP-3, MMP-13, A Disintegrin and Metalloproteinase with Thrombospondin motifs (ADAMTS)-4, and ADAMTS-5. This process may involve intermediate molecules such as NO, cyclooxygenase-2 (COX-2), and prostaglandin E2 (PGE2). Figure created with the help of Figdraw (https://www.figdraw.com).

OA inflammation is a complex pathophysiological phenomenon involving various cell and tissue types both inside and outside the joint. Limited temporal and spatial inflammation is beneficial and necessary for tissue repair and the restoration of homeostasis. However, uncontrolled and dysregulated inflammation is destructive, and forms the basis of chronic inflammation [54]. Additionally, knee pain in OA patients is believed to be associated with local chronic inflammation in the knee joint, involving the production of inflammatory factors such as tumor necrosis factor alpha (TNF-α), IL-6, and nerve growth factor (NGF), which are generally considered to promote pathological OA [55]. Cytokines and chemokines are small proteins secreted by immune cells and other cell types. There are two types of cytokines: pro-inflammatory, including IL-1β, IL-6, IL-7, IL-15, IL-17, IL-18, TNF-α, and anti-inflammatory, including IL-4, IL-8, and IL-10 [56]. We mainly discuss the pro-inflammatory cytokines IL-1, TNF-α, and IL-6 (Fig. 2).

IL-1 is the most classic inflammatory modulator, and one of the earliest studied interleukin markers in OA. IL-1 can upregulate the expression of MMPs in chondrocytes, synovial cells, and macrophages [57]. IL-1 also inhibits the anabolic mechanisms in cartilage tissue by suppressing the expression of type II collagen and proteoglycans in chondrocytes [58]. Under normal conditions, the expression levels of IL-1 in synovial cells and chondrocytes are low, but they can significantly increase in response to inflammatory stimuli. Haynes et al. found that when they cultured synovial cells and chondrocytes from the knee joints of OA patients, the production of IL-1α and IL-1β was elevated to varying degrees [59]. TNF-α is an inflammatory cytokine produced by macrophages during acute inflammation. It is responsible for multiple intracellular signaling events, leading to necrosis or apoptosis [60]. TNF-α can selectively inhibit cartilage collagen production, suppress proteoglycan synthesis, and promote its degradation, which has a certain relationship with OA cartilage destruction and synovitis [61]. Studies also indicate that TNF-α may induce synovial cells and chondrocytes to secrete IL-1β, thereby playing a synergistic role with IL-1β in the pathogenesis of OA. TNF-α can selectively inhibit the production of cartilage collagen, and suppress proteoglycan synthesis while promoting its degradation. Lawrence et al. found that during the early stages of OA, the expression of IL-1 and TNF-α is elevated, and the interaction between IL-1 and TNF-α causes cartilage destruction, leading to the development of knee OA [62]. Studies have shown that traumatic OA is characterized by macrophage and T cell infiltration in the synovium, which subsequently promotes the formation of new blood vessels and accelerates the secretion of inflammatory mediators, thereby exacerbating synovitis. In these processes, the levels of TNF-α are significantly elevated [63]. IL-6 is a crucial cytokine responsible for cartilage destruction, as it can induce inflammatory responses, inhibit chondrocyte proliferation, promote the production of MMPs, and suppress the synthesis of proteoglycans [64]. It has been demonstrated that IL-6 promotes the degradation of cartilage ECM by inducing MMPs expression, resulting in cartilage damage and degeneration [65]. In a study on the epigenetic modification of IL-6 in synovial fibroblasts of OA patients, it was found that IL-6 expression was elevated in these fibroblasts. Inhibiting IL-6 expression in synovial fibroblasts could alleviate OA symptoms, suggesting a link between IL-6 and the pathogenesis of OA [66].

### 3.5 Other effector molecules

The cornerstone of OA is an imbalance between tissue degradation and regeneration. Therefore, this review focused on the degradation and formation of collagen and proteoglycans, such as CTX-II, COMP, and MMPs. Meanwhile, OA is also understood as a disease affecting the entire joint, including various anatomical structures within and surrounding the joint capsule [67]. Consequently, the formation of osteophytes, subchondral bone sclerosis, synovial hyperplasia, and inflammatory infiltration are widely recognized as hallmarks of OA [68]. These distinct pathways collectively offer therapeutic targets and research directions for OA, some of which will be comprehensively reviewed here.

CTX-I, a degradation product of type I collagen, was developed as a urinary biomarker and has since become available in serum as well [69]. Early studies identified a strong spearman correlation between the Kellgren–Lawrence (KL) grade and urinary CTX-I, which might be due to the fact that CTX-I is a marker of bone resorption [69]. Recent study indicated that short-term fluctuations in serum CTX-I and urinary CTX-Iβ might serve as potential biomarkers for predicting the progression of bone marrow lesions in subchondral bone on MRI [70]. Meanwhile, total joint replacement, currently the only definitive treatment for OA, was shown in studies to correlate closely with serum CTX-I levels, which were strongly associated with the risk of knee or hip replacement within the following 2 years [71]. However, some studies analyzing CTX-I levels across different radiographic stages of knee OA found that CTX-I levels did not correlate with the radiographic severity of knee OA [72].

Hyaluronan (HA) is a widely expressed glycosaminoglycan essential for maintaining the structural and functional integrity of articular cartilage. Additionally, it contributes significantly to the unique viscoelastic properties of synovial fluid [73]. One of the changes observed in OA cartilage is an alteration in the typical interaction between HA and proteoglycans [74]. Early study found that serum HA levels were significantly higher in OA group compared with control group, while these levels showed no significant correlation with Western Ontario and McMaster Universities Arthritis Index scores or radiographic OA grading [75]. However, after increasing the sample size, researchers found that serum HA levels were significantly higher in the severe and moderate knee OA groups compared to the normal group, and correlated with the radiographic severity of knee OA [76]. At the same time, a prospective cohort study over 5 years also found that serum HA concentration was correlated with KL grading and joint space narrowing, suggesting that serum HA could be a predictor of knee OA progression [77]. In addition to knee OA, serum HA was also a risk factor for hand OA incidence and was closely associated with both the number of affected joints and the Kallman score [78].

C-reactive protein (CRP) is a central component of the innate immune inflammatory response, with its synthesis primarily driven by pro-inflammatory factors released by macrophages and adipocytes [79]. Compared to control group, systemic CRP levels in OA patients were significantly elevated and were correlated with clinical features and the radiographic severity of OA [80]. A study involving 845 participants found that serum CRP levels were slightly elevated in early knee OA, while higher serum CRP levels indicated that OA was likely to persist for more than 4 years [81]. However, other studies showed that, after adjusting for BMI, serum CRP levels were not associated with the prevalence of knee OA [82]. Likewise, a 5-year prospective follow-up study found no significant relationship between CRP levels and the incidence of knee or hip OA after adjusting for BMI [83]. This finding suggested that the increase in knee OA prevalence was largely due to rising BMI. In a recent study, researchers fed hCRP-transgenic male mice a 45 % high-fat diet for 38 weeks and observed degeneration of cartilage and bone hyperplasia. Thus, CRP was considered an independent factor that exacerbated the development of HFD-induced OA [84].

## 4 Biomarkers (from bioinformatics)

**Fig. 3:**
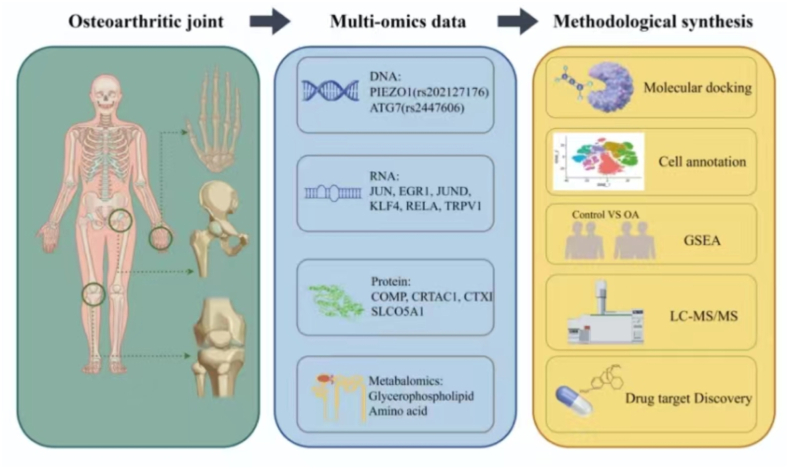
**The applications of proteomics, metabolomics, genomics, transcriptomics, and multi-omics in OA**. Figure created with the help of Figdraw (https://www.figdraw.com).

Nowadays, omics technologies, such as proteomics and metabolomics, are frequently integrated into the routine methodologies of biological researchers [85]. Concurrently, bioinformatics techniques are increasingly applied in OA. Thus, this review focuses on studies on the application of proteomics, metabolomics, genomics, transcriptomics, and multi-omics in OA, as illustrated in Fig. 3.

### 4.1 Proteomics

Proteomics, a field at the intersection of biology and chemistry, focuses on the comprehensive study of proteins within a cell, tissue, or organism [86]. Traditional methods often rely on indirect measurements through enzyme activity. In contrast, utilizing liquid chromatography-tandem mass spectrometry (LC-MS/MS), proteomics can identify and quantify thousands of proteins from smaller sample volumes, making it ideal for high-throughput analysis of complex samples like serum [87]. Thus, proteomics has the potential to identify specific markers for early diagnosis of OA.

Proteomic analyses in OA began around 2004. However, these studies faced significant challenges due to technical limitations, particularly in terms of sensitivity and throughput. With the advancement of technology, new large-scale affinity proteomics platforms, such as the aptamer-based SomaScan platform and the proximity extension assay developed by Olink, have been created to advance biomarker discovery and risk prediction. Using the SomaScan platform, researchers measured 4792 proteins in the plasma of 39,155 individuals to identify potential biomarkers for hip, knee, and hand OA. They believe that CRTAC1 may be a novel OA candidate biomarker because it has the highest correlation with OA diagnosis, and can predict the progression to joint replacement [88]. Meanwhile, in a recent study using the Olink platform, the levels of 184 plasma proteins were measured in 3517 participants, and machine learning was employed to explore the relationship between these protein levels and the severity and progression of knee, hip, and hand OA. The study identified 8 proteins significantly associated with the overall OA burden, including CRTAC1 and COMP. By constructing a multivariable regression model, researchers also concluded that CRTAC1 is the most convincing and reliable biomarker for OA severity and progression [89]. Compared to plasma samples, urine sample collection is non-invasive, but its low protein concentration and high variability present challenges for proteomic analysis [90]. Henrotin et al. conducted proteomic analysis on urine samples from 10 women, identifying 13 proteins with significant intergroup differences. They focused on two peptides of fibrinogen, naming them Fib3-1 and Fib3-2, and validated their presence in serum samples using Enzyme-linked immunosorbent assay (ELISA) [91]. To further illustrate the effectiveness of multi-sample proteomics in screening for OA biomarkers, Kraus et al. conducted a cohort study on an OA population. By measuring the serum and urine of 194 OA patients and integrating clinical information to construct a multivariable predictive model, they identified eight biomarkers, including serum CTXI, serum HA, and serum NTXI. The model demonstrated high accuracy with an AUC value of 0.667 [92]. Unlike serum and urine samples, synovial fluid is in direct physical contact with synovium, ligaments, menisci, joint capsule, and bones. During OA, structural and metabolic changes in any of these tissues should be reflected in the proteomic composition of synovial fluid. Therefore, proteomic studies of synovial fluid are crucial for understanding OA [93]. A proteomic analysis comparing synovial fluid from OA and non-OA knee joints revealed that 70 proteins had relatively high abundance in OA synovial fluid. They also found that SLPI, C8, CLU, FN1, RARRES2, MATN3, and MMP3 exhibited tissue-dependent expression, suggesting that these proteins could serve as new tissue-specific candidates for developing OA biomarkers [94]. By measuring synovial fluid samples from healthy or mildly degenerated individuals and knee replacement patients using the SOMAscan platform, researchers estimated the abundance differences of 6251 proteins among the three groups. The results revealed that 583 proteins were upregulated in late-stage OA, including MMP1, MMP-13, and IL-6. Furthermore, the experimental data demonstrate that biomarkers for early OA can be identified by combining proteomics with Gaussian Graphical Models [95]. In addition to biomarker screening studies, proteomics can also be used to screen for drug action targets. To evaluate the efficacy of the nonsteroidal anti-inflammatory drug celecoxib on OA, proteomic analysis and ELISA validation were performed on plasma samples from patients taking the medication, identifying LGALS1, LGALS3, and CD44 as potential biomarkers for assessing the clinical response of OA patients to celecoxib [96].

### 4.2 Metabalomics

Increasing evidence suggests that the onset and progression of OA should be attributed to a combination of multiple factors, including chronic low-grade inflammation, obesity, hyperglycemia, and unhealthy lifestyles. Therefore, a new OA phenotype, referred to as “metabolic OA” is gaining increasing attention [97]. Recent studies have shown that OA involves chronic low-grade inflammation and metabolic dysregulation within joint tissues, leading to the degradation of bone and cartilage [98]. Metabolomics refers to the comprehensive and dynamic changes in metabolic processes within biological systems due to biological stimuli, pathophysiological disruptions, or alterations in genetic information [99]. Thus, using metabolomics to study OA holds significant potential.

The first application of metabolomics in OA was around 2009, using Nuclear Magnetic Resonance (NMR)-based metabolomics to explore the cartilage degradation process in guinea pigs with OA [100]. With the development of technology, metabolomics mainly focuses on the influence of metabolite types on OA. In terms of the application of amino acid metabolism in OA, amino acids play a critical role in energy production, cell signaling, and maintaining metabolic homeostasis [101]. Research on synovial fluid has found that OA subjects exhibit significant changes in glycerophospholipid metabolism, sphingolipids, amino acid metabolism, leukotriene/arachidonic acid metabolism, glutathione metabolism, and tricarboxylic acid (TCA) cycle activity [102]. Metabolomic analysis of subchondral bone by Swank et al. indicates alterations in sphingolipid metabolism, purine/pyrimidine metabolism, and amino acid metabolism in OA patients [103]. These studies suggest that the direct metabolic environment of OA joints is characterized by changes in energy utilization, oxidative stress, and inflammatory states. Meanwhile, an increasing number of studies support the evidence that amino acid metabolism is associated with various types of pain, such as chronic back pain, abdominal pain, joint pain, and neuropathic pain [104]. Therefore, by measuring metabolites and cytokines in the serum samples of patients, significant correlations were found between pain scores and acylcarnitine, carnosine, cortisol, cortisone, cystine, dopamine, coursodeoxycholate sulfate, phenethylamine, and succinate. Additionally, inflammatory factors IL-10, IL-13, IL-1β, IL-2, IL-8, and TNF-α were found to be associated with these key metabolites. These metabolites can serve as biomarkers for the development of new therapeutic drugs to alleviate knee pain and treat OA [105].

In terms of the application of lipid metabolism in OA, lipid metabolism refers to the process of digestion, absorption, synthesis, and breakdown of fats in the body [106]. Increasing evidence suggests that lipid accumulation in chondrocytes is an important factor influencing the occurrence and progression of OA. Dysregulated lipid metabolism leads to lipid accumulation, accelerating cartilage degradation and resulting in the upregulation of cartilage-degrading enzymes [107]. Choi et al. identified the CH25H-CYP7B1-RORα axis of cholesterol metabolism in chondrocytes as a key catabolic regulator in the pathogenesis of OA. This axis primarily contributes to increased cholesterol levels in osteoarthritic chondrocytes by upregulating cholesterol hydroxylases (CH25H and CYP7B1) and increasing the production of oxysterol metabolites. However, knocking out or inhibiting these hydroxylases was found to suppress the pathogenesis of OA. Consequently, the researchers suggest that targeting the CH25H-CYP7B1-RORα axis in cholesterol metabolism could offer a therapeutic approach for treating OA [108]. Additionally, the metabolic pathways of chondrocytes are closely related to cellular aging [109]. Research has found that CircRREB1 participates in chondrocyte aging and age-related OA by regulating FASN-associated lipid metabolism. Therefore, targeting CircRREB1 is a potential method for treating age-related OA [110]. In summary, despite the differences in specific metabolites reported in these studies, common metabolic themes emerged in many reports. Some studies identified metabolites related to mitochondrial dysfunction, such as alterations in fatty acid metabolism [111] and TCA cycle metabolites [112]. Additionally, certain studies found metabolites indicating the presence of reactive oxygen species, gluconeogenic amino acids, and antioxidants [113]. Future research could expand these findings by focusing on metabolites associated with mitochondrial dysfunction.

### 4.3 Genomics

The purpose of genomics research is to identify genetic variations that affect the risk of disease [114]. The development of innovative genetic technologies has continually improved the genomic resolution of large-scale analyses. Advances in computational and statistical sciences have made significant contributions to the success of genetic epidemiology. The Human Genome Project and the HapMap Project, initiated in the early 1990s, mapped single nucleotide polymorphisms (SNPs) associated with diseases and treatment responses [115]. The publication of the first genome-wide association study (GWAS) report in 2005 marked a new era in genetic epidemiology [116]. In 2006, the first genome analysis report on OA was published, analyzing the genomes of 1891 individuals from three populations. It was found that the LRCH1 gene variant (rs912428) was consistently associated with knee OA across the three populations [117]. Many more OA GWASs followed thereafter, with increasing sample size allowing for the robust detection of reproducible signals, primarily in Caucasian and Asian populations. GWASs of African populations are still rare. One of the few was performed in African Americans. GWAS of knee OA was conducted on 1217 African Americans from two North American cohort studies. The results revealed the LINC01006 gene, which is less common among European Americans. Moreover, pathway analysis revealed that the dorsal/ventral neural tube patterning and iron ion transport pathways were significantly associated with knee OA in African Americans. These findings highlight the genetic differences in knee OA between African Americans and European Americans, underscoring the necessity of including diverse populations in OA genetic research [118]. Consequently, a large GWAS analysis on OA was conducted, involving 826,690 individuals (177,517 OA patients) from 13 international cohorts across 9 populations. The study identified 100 independent risk variants associated with 11 OA phenotypes, demonstrating that the genetic risk for OA varies depending on the joint site. It also revealed genetic correlations between OA and pain-related phenotypes and identified signal enrichment in neural pathways [119].

Although many loci have been mapped to the “nearest gene”, it is important to note that due to the three-dimensional structure of chromosomes, SNPs can also affect distant genes. Therefore, understanding the precise role of genetic variation in OA susceptibility has always been challenging, which is essential for translating genetics into clinical practice [120]. Hence, in a GWAS focusing on the hip joint, researchers aimed to explore the genetic structure of cam morphology. They used the alpha angle as a proxy measurement for cam morphology. Through a Mendelian randomization approach, they conducted a GWAS for the alpha angle, assessing its causal relationship with hip OA. The researchers found that the alpha angle has a causal risk effect on hip OA, while genetic predisposition for hip OA had stronger evidence of a causal effect on increased alpha angle. This indicates a bidirectional relationship between the alpha angle and hip OA. Therefore, the researchers suggested that genetic susceptibility to hip OA could help in understanding the relationship between hip OA in the elderly and cam morphology [121]. In the study exploring the genetic associations of OA and joint replacement therapy, data from over 700,000 individuals, including knee and hip OA cases and healthy controls, were collected, identifying 52 genetic variants associated with OA. More importantly, surgical phenotype analysis revealed 10 novel variants, including genes related to mechanotransduction (PIEZO1: rs202127176) and autophagy (ATG7: rs2447606). The findings indicate that the genetic correlation of knee and hip OA varies depending on the status of joint replacement surgery [122].

Pain is a major cause of disability worldwide, imposing a significant burden on individual health and society. It has been shown that genetic variations are associated with pain in OA patients [123]. In a study of 171,516 participants from the UK Biobank, two genetic variants associated with knee pain were identified: GDF5 (rs143384) and COL27A1 (rs2808772). The findings were supported by two independent OA replication cohorts within the same study [124]. Meanwhile, by collecting blood samples from OA patients and conducting whole-exome sequencing, significant differences were found in 507 gene regions between two groups of patients. Genes such as CASP5, RASGEF1A, and CYP4B1 showed notable differences between the groups [125].

### 4.4 Transcriptomics

Transcriptomics is a field of study that involves the analysis of all RNA transcripts produced by the genome of a cell or organism at a specific time [126]. The development of high-throughput technologies has deepened the understanding of OA pathophysiology at the tissue, cellular, and molecular levels. RNA sequencing was used to analyze the differential gene expression in knee cartilage tissues from 18 healthy individuals and 20 OA patients, identifying key transcription factors such as JUN, EGR1, JUND, KLF4, RELA, and FOS. Meanwhile, 15 pathways were identified as significantly disrupted in OA, with the most notably affected pathways being those related to the ECM, PI3K-Akt, HIF-1, FoxO, and circadian rhythm [127]. In the study of genetic differences in meniscus tissue between OA patients and non-OA individuals, CSN1S1, COL10A1, and WIF1 genes were identified as playing a key role in OA patients. These genes are potential biomarkers for OA [128]. Furthermore, in the transcriptome study of subchondral bone in OA, unsupervised hierarchical clustering identified IL-11 and CHADL as strong potential therapeutic targets for OA [129]. In addition to RNA-seq, circular RNA, a class of non-coding RNA with a circular structure, is involved in various important pathophysiological processes of OA, especially through its competing endogenous RNA mechanism, playing a significant role in OA [130]. Although RNA-seq has many applications in OA, due to practical reasons, this technology is usually performed on tissue samples containing thousands to millions of cells, which hinders the direct assessment of the fundamental unit of biology—the single cell [131]. However, with the development of sequencing technologies, the invention of single-cell RNA sequencing (scRNA-seq) can fill some gaps caused by bulk RNA sequencing [132]. Here, we emphasize the application of scRNA-seq in different joint tissues affected by OA.

The application of scRNA-seq in OA includes its use with chondrocytes, synovial cells, and meniscus cells. From the perspective of cartilage, Ji et al. conducted scRNA-seq analysis on OA chondrocytes, identifying 1464 chondrocytes and defining 7 chondrocyte subgroups. This revealed the intrinsic states of chondrocytes and identified potential transitions among proliferative chondrocytes, pre-hypertrophic chondrocytes, and hypertrophic chondrocytes. They also identified new markers for chondroprogenitor cells (CPCs) and, through computational analysis, demonstrated the relationship between CPCs and fibrocartilage cells [133]. In addition to research on knee cartilage subgroups, scRNA-seq analysis was performed on samples from 5 donors to explore the characteristics of hand OA. This analysis identified 13 chondrocyte subgroups among 105,142 chondrocytes, discovering a new subgroup with inflammatory regulation properties. Compared to knee OA, hand OA exhibits characteristics of ferroptosis and is associated with the key target FTH1. Furthermore, Mendelian randomization and community studies confirm that the FTH1 target can regulate ferroptosis in OA chondrocytes [134]. Similarly, by comparing scRNA-seq levels of the knee joints of healthy individuals and OA patients, it was found that TRPV1 plays a protective role in chondrocyte ferroptosis. Furthermore, pharmacological activation of TRPV1 significantly reduced cartilage degeneration by protecting chondrocytes from ferroptosis [135]. This suggests that different therapeutic targets may exist for OA chondrocytes in different locations. From the perspective of the synovium, the molecular crosstalk between cartilage and synovium has been elucidated, identifying 12 different synovial cell types and 7 distinct chondrocyte types. It was discovered that the primary contributors to OA are inflammatory macrophages and dendritic cells. These cells produce classic pathogenic factors such as IL-1β, which influences OA progression [136]. Further investigating the role of synovium in OA, Tang et al. discovered that dipeptidyl peptidase $4^{+}$ mesenchymal cells are common progenitor cells for infrapatellar fat pad (IPFP) adipocytes and synovial lining fibroblasts, suggesting IPFP and synovium represent an integrated tissue unit. They identified that the Apolipoprotein E (APOE) signaling from intermediate fibroblasts and macrophages is a key regulatory factor, as inhibiting APOE signaling through intra-articular injection of anti-APOE neutralizing antibody could attenuate the progression of collagenase-induced OA in mice [137]. From the perspective of meniscus, a new mechanism of meniscus degeneration was discovered. Single-cell sequencing was conducted on healthy and degenerated human meniscus cells, identifying 7 cell subgroups. It was demonstrated that CD146+ meniscus cells are progenitor cells, and it was found that IL1β-induced degeneration of meniscus progenitor cells is a potential mechanism leading to meniscus degeneration [138].

### 4.5 Multi-omics

Multi-omics typically refers to the integrated application of various high-throughput screening technologies such as genomics, transcriptomics, proteomics, and metabolomics. This integration is necessary because single-omics technologies cannot fully realize their potential in disease research. In practice, a single-omics study can only perform correlation analysis with diseases, primarily reflecting changes in the disease process without explaining causal relationships. For example, in OA, even if a biochemical molecule is statistically related to the disease, it cannot elucidate the complex mechanisms behind the disease. However, by integrating multiple omics, scientists can identify new associations between biomolecules and disease phenotypes, recognize relevant signaling pathways, and establish detailed disease biomarkers [139].

To investigate the mechanism of ferroptosis in aging chondrocytes, a combined analysis of metabolomics and single-cell transcriptomics was conducted. The study found that although aging chondrocytes exhibit excessive activation of ferroptosis metabolism, this can be counteracted by the overexpression of EAAT1. EAAT1 increases intracellular glutamate levels and activates the glutathione system, thereby combating ferroptosis. Consequently, researchers propose that EAAT1 could serve as an effective and specific target for anti-aging therapy in OA [140]. To obtain a comprehensive molecular profile of cartilage degeneration, Julia et al. analyzed the whole-genome, transcriptomics, and quantitative proteomics data of chondrocytes from 38 patients. The study identified 49 genes differentially regulated between intact and degraded cartilage, including AQP1, COL1A1, and CLEC3B. Pathway analysis revealed that ECM degradation, collagen catabolism, and angiogenesis are involved in the progression of OA [141]. To obtain a comprehensive molecular profile of the synovium, genomic and transcriptomic analyses were conducted on synovial and blood samples from 245 OA patients. Researchers identified 4765 primary and 616 secondary expression quantitative trait loci (eQTLs) in the synovium. By integrating GWAS and eQTL data, they identified 84 arthritis-related genes, such as JAZF1, and discovered 38 new genes that had not been previously reported using GTEx data or immune cell eQTL studies, including IL2RA, ERBB2, and RRK2 [142]. In addition to disease target screening, multi-omics is also useful in drug target identification. Chen et al. aimed to explore the potential drug mechanism of Guizhi Decoction (GZD) for treating OA. They used ultra-performance liquid chromatography to identify the active components in GZD and then combined this with transcriptomics to screen for differential targets before and after drug treatment. The study ultimately found that in mice treated with GZD, mRNA levels of TNF-α, IL-1β, IL-6, MMP3, and MMP9 were downregulated. The researchers inferred that the anti-OA mechanism might be related to modulating the TNF signaling pathway and inhibiting the inflammatory response [143]. Franco-Trepat et al. explored the effects of β-boswellic acid (BBA) on OA. Using computational pharmacology, proteomics, transcriptomics, and metabolomics, they analyzed the therapeutic differences of BBA in mice and primary human OA joint cells. They found that BBA binds to the TLR4 complex and inhibits TLR4 and IL1R signaling in OA chondrocytes, osteoblasts, and synovial cells [144]. In summary, the study of biological multi-omics data systematically reveals the physiological or pathological molecular profiles of OA. However, although multi-omics can provide data from different dimensions, the various omics technologies have differing levels of precision. The signal-to-noise ratio in multi-omics measurements often affects the integration of multi-omics data.

## 5 Summary

In cytokines and inflammation-related biomarkers, it has been observed that levels of IL-6, IL-8, IL-1, IL-1 receptor antagonist, IL-1β, VEGF, and CRP elevate immediately following joint injury and subsequently decrease over time [145,146]. In ECM-related biomarkers, the concentrations of the collagenase-cleaved type I and II collagen neoepitope, COMP, and CTX-II show increasing levels as OA progresses [12,25,147]. In the category of matrix protease-related biomarkers, MMP-3, MMP-9, MMP-13, neutrophil elastase, and tissue inhibitor of metalloproteinase-1 increase in response to mechanical stress [148,149]. Therefore, careful monitoring of changes in these biomarkers could theoretically reflect the progression of OA. However, no biomarkers have yet been identified to reliably diagnose OA or predict its prognosis. This may be because, although biomarkers can demonstrate sensitivity and specificity in detecting certain treatment effects, their selection must be aligned with the specific objectives and desired outcomes of the study [150]. For example, changes in the levels of some biomarkers like MMP-13 and COMP are also observed in other systemic diseases, such as rheumatoid arthritis [151], autoimmune disorders [152], and liver cirrhosis [153].

An ideal biomarker should be non-invasive, detectable at the early stages of disease, and related to prognosis. It should also be specific to a particular tissue type and closely connected to the pathophysiology of the disease [154]. OA, being a multifactorial disease, involves various factors such as trauma, immune response, metabolic changes, and inflammatory processes. There is currently no perfect OA biomarker, largely because of the inherent complexity of OA. The application of proteomics, metabolomics, genomics, and transcriptomics facilitates a comprehensive understanding of OA pathology across multiple biological scales, including the molecular, cellular, and tissue levels. Integrating multi-omics techniques at the individual level allows us to explore the interactions across different omics layers, offering critical molecular insights that inform the diagnosis, treatment, and prognosis of OA. Although multi-omics currently has limitations, such as the difficulty in obtaining matching datasets for different omics layers or consistent data from the same experiments [155], integrated multi-omics analysis can offer a deeper understanding of the mechanisms behind OA progression compared to single-omics analysis.

In this review, we describe studies that employ commonly used OA biomarkers, explore the application of four omics approaches to OA, and investigate OA biomarkers through multi-omics integration. Looking ahead, as multi-omics methods advance and research delves deeper into the molecular and genetic mechanisms behind OA pathogenesis, new therapeutic targets and personalized treatments will be developed.

## Authors’ contributions

M.D. contributed to the literature review, initial draft and manuscript editing. C.T. contributed to literature review, initial draft and figure preparation. Y.L. contributed to the literature review and manuscript editing. Y.J. contributed to the literature review and manuscript editing. Y.H. contributed to literature review and manuscript editing. Y.F. contributed to manuscript editing. C. C. contributed to the literature review and drafted and edited the manuscript. All the authors provided final approval.

## Data availability

Data sharing is not applicable to this article as no new data were created or analyzed in this study.

## Funding

This study was supported by the 10.13039/501100005230Natural Science Foundation of Chongqing (cstb2022nscq-msx0139), and the Future Medical Innovation Team of 10.13039/501100004374Chongqing Medical University (W0080).

## Declaration of competing interest

The authors declare that they have no known competing financial interests or personal relationships that could have appeared to influence the work reported in this paper.
